# The effect of a cognitive training therapy based on stimulation of brain oscillations in patients with mild cognitive impairment in a Chilean sample: study protocol for a phase IIb, 2 × 3 mixed factorial, double-blind randomised controlled trial

**DOI:** 10.1186/s13063-024-07972-7

**Published:** 2024-02-23

**Authors:** Alejandra Figueroa-Vargas, Begoña Góngora, María Francisca Alonso, Alonso Ortega, Patricio Soto-Fernández, Lucía Z-Rivera, Sebastián Ramírez, Francisca González, Paula Muñoz Venturelli, Pablo Billeke

**Affiliations:** 1 Laboratorio de Neurociencia Social y Neuromodulación del Centro de Investigación en Complejidad Social (neuroCICS), Facultad de Gobierno, Universidad del Desarrollo, Santiago, Chile; 2 Centro de Investigación del Desarrollo en Cognición y Lenguaje (CIDCL), Universidad de Valparaíso, Valparaíso, Chile; 3 Centro de Estudios Clínicos, Facultad de Medicina, Clínica Alemana-Universidad del Desarrollo, Santiago, Chile; 4 Laboratorio LaNCE, Centro Interdisciplinario de Neurociencia, Facultad de Medicina, Pontificia Universidad Católica de Chile, Santiago, Chile

**Keywords:** Mild cognitive impairment, Cognitive training, Working memory, Cognitive functions, Non-invasive brain stimulation, Randomised controlled trial

## Abstract

**Background:**

The ageing population has increased the prevalence of disabling and high-cost diseases, such as dementia and mild cognitive impairment (MCI). The latter can be considered a prodromal phase of some dementias and a critical stage for interventions to postpone the impairment of functionality. Working memory (WM) is a pivotal cognitive function, representing the fundamental element of executive functions. This project proposes an intervention protocol to enhance WM in these users, combining cognitive training with transcranial electrical stimulation of alternating current (tACS). This technique has been suggested to enhance the neuronal plasticity needed for cognitive processes involving oscillatory patterns. WM stands to benefit significantly from this approach, given its well-defined electrophysiological oscillations. Therefore, tACS could potentially boost WM in patients with neurodegenerative diseases.

**Methods:**

This study is a phase IIb randomised, double-blind clinical trial with a 3-month follow-up period. The study participants will be 62 participants diagnosed with MCI, aged over 60, from Valparaíso, Chile. Participants will receive an intervention combining twelve cognitive training sessions with tACS. Participants will receive either tACS or placebo stimulation in eight out of twelve training sessions. Sessions will occur twice weekly over 6 weeks. The primary outcomes will be electroencephalographic measurements through the prefrontal theta oscillatory activity, while the secondary effects will be cognitive assessments of WM. The participants will be evaluated before, immediately after, and 3 months after the end of the intervention.

**Discussion:**

The outcomes of this trial will add empirical evidence about the benefits and feasibility of an intervention that combines cognitive training with non-invasive brain stimulation. The objective is to contribute tools for optimal cognitive treatment in patients with MCI. To enhance WM capacity, postpone the impairment of functionality, and obtain a better quality of life.

**Trial registration:**

ClinicalTrials.gov NCT05291208. Registered on 28 February 2022. ISRCTN87597719 retrospectively registered on 15 September 2023.

**Supplementary Information:**

The online version contains supplementary material available at 10.1186/s13063-024-07972-7.

## Administrative information

Note: the numbers in curly brackets in this protocol refer to the SPIRIT checklist item numbers. The order of the items has been modified to group similar items (see http://www.equator-network.org/reporting-guidelines/spirit-2013-statement-defining-standard-protocol-items-for-clinical-trials/).

**Table:** 

Title {1}	The effect of a cognitive training therapy based on stimulation of brain oscillations in patients with Mild Cognitive Impairment in a Chilean sample: study protocol for a randomised controlled trial.
Trial registration {2a and 2b}.	ClinicalTrials.gov NCT05291208. 28.02.2022 ISRCTN87597719 15.09.2023
Protocol version {3}	Version 2.0 [10.17.2022]
Funding {4}	This trial is funded through the National Fund for Health Research and Development in Health, Chile (FONIS for its acronym in Spanish). The funder’s number for this trial is SA19I0118.
Author details {5a}	Alejandra Figueroa-Vargas, Universidad del Desarrollo, Faculty of Government, Av. Las Condes #12,461 office 307, Las Condes, Chile. Phone: + 56 9 9819 4860, email: amfigueroa@udd.cl Pontificia Universidad Católica, Faculty of Medicine, Lira #40, Santiago, Chile. Phone: + 56 2 2354 2000, email: abfigueroa@uc.cl Begoña Góngora, Universidad de Valparaíso, Faculty of Medicine, Angamos 655, Viña del Mar, Chile. Phone: + 56 32 250 7695, email: begona.gongora@uv.cl Francisca Alonso, Universidad de Valparaíso, Faculty of Medicine, Angamos 655, Viña del Mar, Chile. Phone: + 56 32 250 7695, email: mariafrancisca.alonso@uv.cl Alonso Ortega, Universidad de Valparaíso, Faculty of Medicine, Angamos 655, Viña del Mar, Chile. Phone + 56 32 250 7695, email: alonso.ortega@uv.cl Lucía Z-Rivera, Universidad de Valparaíso, Faculty of Medicine, Angamos 655, Viña del Mar, Chile. Phone + 56 32 250 7695, email: luciaa.z.r@gmail.com Sebastián Ramírez, Universidad de Valparaíso, Faculty of Medicine, Angamos 655, Viña del Mar, Chile. Phone + 56 32 250 7695, email: ramirezselame@gmail.com Patricio Soto-Fernández, Universidad de Valparaíso, Faculty of Medicine, Angamos 655, Viña del Mar, Chile. Phone + 56 32 250 7695, email: patricio.soto@uv.cl Francisca González, Clínica Alemana—Universidad del Desarrollo, Faculty of Medicine, Av. Plaza 680, Las Condes, Chile. Phone: + 56 2 2327 9110 email: frgonzalez@udd.cl Paula Muñoz-Venturelli, Clínica Alemana -Universidad del Desarrollo, Faculty of Medicine, Av. Plaza 680, Las Condes, Chile. Phone: + 56 2 2327 9110, email: paumunoz@udd.cl Pablo Billeke, Universidad del Desarrollo, Faculty of Government, Av. Las Condes #12,461 office 307, Las Condes, Chile. Phone: + 56 9 9925 9790 Email:pbilleke@udd.cl
Name and contact information for the trial sponsor {5b}	Vicerectorate of Research and Doctoral Programs, Universidad del Desarrollo, Santiago, Chile. Marie Denise Saint-Jean Matzen, Director of Office of Research and Doctoral Programs. Av. Plaza 680, Las Condes, Chile. Phone: + 56 2 2327 9110 Email: dsaintjean@udd.cl
Role of sponsor {5c}	The trial sponsor has ultimate authority over the management of the study. Neither the funder nor the trial's sponsor was involved in the study’s design and will not be involved in the collection, analysis, or interpretation of data or the writing of the study report and the decision to submit it for publication.

## Introduction

### Background and rationale {6a}

Globally, sociodemographic changes reflect progressive population ageing. By 2050, the World Health Organization (WHO) projects that more than one in five people will be over 60, with 80% residing in low- and middle-income countries [1, 2]. This shift has increased the prevalence of chronic diseases, notably dementia, affecting cognitive, psychological, and behavioral functions, thereby challenging individuals, families, and public policies [3–5].

Conventional drug treatments for dementia have shown limited long-term efficacy, prompting the suggestion of non-pharmacological therapies as complementary treatments, particularly in the early stages like mild cognitive impairment (MCI) [6–8]. Working memory (WM), a vital cognitive component, has been identified as a modifiable factor through training, with evidence suggesting improved capacity and brain activity [9–11]

In neurodegenerative diseases, limited neuroplasticity poses challenges, but non-invasive brain stimulation (NIBS) could offer a promising avenue for enhancing cognitive rehabilitation. Integrating these advances into early intervention and cognitive training programmes for MCI patients remains an unexplored area.

#### Mild cognitive impairment (MCI)

MCI is clinically diagnosed based on observed cognitive changes, altered cognitive domains, independent daily living, and preserved functional and social performance [12]. This condition is considered a precursor to dementia, with specific profiles predicting the likelihood of different types of dementia. Amnestic MCI indicates a higher risk of developing Alzheimer’s disease, while a disjunctive predominance suggests frontotemporal dementia [13].

#### MCI and cognitive intervention

Studies have indicated the potential preservation of cognitive abilities and the partial reversibility of impairments in patients with MCI [14]. Environmental and lifestyle factors, including education, professional and leisure activities, and experiences, have been found to influence cognitive maintenance [15]. Considering the preventive role of cognitive training, it is reasonable to view it as a potential therapeutic tool. Improved performance has been observed in trained functions among subjects with single- and multiple-domain MCI [16]. Additionally, neurophysiological studies have shown alterations in brain oscillations in MCI patients similar to those seen in dementia patients [17].

#### MCI and non-invasive brain stimulation

Studies have demonstrated that altered brain oscillatory patterns underlie cognitive impairments in dementia [18, 19]. NIBS therapies can target these patterns, enhancing residual cognitive capacity at the early stage of impairment. Transcranial electrical stimulation, a validated and safe intervention, induces neuronal plasticity without directly triggering action potentials [20]. Among these techniques, transcranial direct current stimulation (tDCS) enhances excitability near the anode, while transcranial alternating current stimulation (tACS) modulates oscillatory activity to boost neuroplasticity [21]. Particularly, tACS offers a precise means of enhancing neuronal plasticity for cognitive processes like working memory (WM), given the detailed understanding of its underlying electrophysiological patterns [22].

#### Working memory (WM) and NIBS

Working memory (WM) is a crucial cognitive capacity facilitating the temporary manipulation of information for future behavioral guidance. Its neural basis involves distributed functional networks primarily in fronto-parietal association cortices, with theta and nested gamma oscillations [23–26]. Alterations in brain oscillatory dynamics, especially in the theta and gamma frequency ranges, have been linked to cognitive impairments in various neurodegenerative diseases, indicative of potential early cognitive reserve loss [18, 19, 27–29].

Implementing WM training programmes has demonstrated improved cognitive performance, attributed to increased activity, connectivity, and selectivity within fronto-parietal networks, potentially explaining the observed transfer effect to enhance daily functionality [11].

#### Cognitive training and NIBS in MCI Patients

To date, no initiatives offering interventions combining cognitive training and neuroplasticity facilitation have been made available for individuals with MCI. Studies have shown the medium-term efficacy of tDCS in enhancing working memory (WM) in the older adult population [30]. However, evidence suggests that tACS may be more efficient than tDCS in improving WM [22]. Clinical trials in progress are investigating the combined impact of tDCS and WM training in MCI [31, 32].

Given the need for effective therapeutic alternatives to enhance the quality of life and reduce cognitive disability, this study proposed to evaluate the effect of a WM training therapy, which involves tACS on the prefrontal oscillatory activity, in a group of users with MCI compared to an intervention involving only cognitive training. Recent research demonstrates the potential of oscillatory electrical stimulation, particularly a specific gamma wave (80 Hz) synchronised with the positive phase of theta oscillation (6 Hz), in enhancing WM and increasing connectivity within brain regions [33]. This evidence supports the notion that brain oscillatory activity can be targeted for cognitive training, fostering neuroplasticity [34].

### Objectives {7}

#### Aims

To evaluate the effect of a combined cognitive training programme (CCTP) that involving cognitive training and transcranial alternating current electrical stimulation on prefrontal oscillatory activity in a group of patients with mild cognitive impairment, compared to a traditional cognitive training programme (TCTP) involving only cognitive training.

##### ***Hypothesis***

We hypothesise that:

1. MCI patients who participate in the combined cognitive training programme (CCTP) present a pre-post-intervention increase in prefrontal theta oscillatory activity, which will be greater than the increase submitted by those patients who participate in the traditional cognitive training programme (TCTP).
2. The increase in prefrontal theta oscillatory activity generated by the combined cognitive training program (CCTP) is reflected in an addition in the cognitive performance post-intervention of patients with MCI concerning their cognitive performance pre-intervention.

### Trial design {8}

This study is a phase IIb randomised, double-blind clinical trial type study. It will be a parallel design where two groups receive two treatments (interventions). We will study the characteristics of the factors and effects. In this manner, this analysis will allow us to conceptualise the design as a 2 × 3 mixed factorial. Thus, we will include a factor between groups (type of intervention, two levels) and an intra-subject factor (measured in three episodes, one pre-intervention, and two post-intervention). Based on the factor between groups, it will implement two different interventions. The treatment group will receive a cognitive training intervention combined with alternating current electrical stimulation (i.e. treatment). The control group will receive a traditional cognitive training intervention with placebo electrical stimulation. Depending on the intra-subject factor, two dependent variable measurements will be implemented: one pre-intervention and one post-intervention measure. This design will allow the study of the potential interaction effects between the inter- and intra-subject factors [35].

## Methods: participants, interventions, and outcomes

### Study setting {9}

The target population will be non-institutionalised patients with MCI referred to the Center of Phonoaudiological Attention of the University of Valparaiso (CAFUV) from Valparaiso, Chile. Assessments will be conducted on site by a clinical team consisting of a neuropsychologist, a clinical psychologist, and speech therapists at the research centre, who will apply the instruments to corroborate the fulfilment of the inclusion–exclusion criteria.

### Eligibility criteria {10}

We will recruit participants diagnosed with mild cognitive impairment (MCI).

*Inclusion criteria* will be:

1. MCI diagnostic, according to the criteria established by Petersen et al.[13].
2. Age equal to or greater than 60 years.
3. Six or more years of complete schooling (presence of reading and writing).

Potential participants will be excluded if:

1. The previous diagnosis of other neurodegenerative diseases.
2. Attending another cognitive training programme.
3. A history of epilepsy or the current presence of epileptic seizures.
4. The presence of psychiatric diseases.
5. The presence of a relevant depressive picture (GDS >  = 2).
6. History of important neurological alterations such as the history of stroke, transient ischemic attack, and cranial brain trauma.
7. Significant alterations of communication.

### Who will take informed consent? {26a}

Informed consent will be obtained through a two-phase process. Participants will first receive information from their clinical team. They will then grant permission for the research team to contact them for an initial discussion of the study and screening process. A study researcher will obtain full informed consent before the eligibility and baseline assessment, which will be conducted in person. Potential participants will receive complete information about the study before the interview, and at the point of consent, there will be additional opportunities to discuss the research and for participants to ask any questions. The option to withdraw from the trial will be fully explained, and researchers will be trained in obtaining informed consent, including assessing capacity to consent where appropriate and supervised by the CI. Consent will only be obtained from individuals capable of making an informed decision regarding their participation.

### Additional consent provisions for collecting and using participant data and biological specimens {26b}

On the consent form, participants will be asked if they agree to be contacted about ethically approved research studies for which they might be suitable. Participants will also be asked if they agree to their anonymised data, anonymised transcripts, and session recordings being used in future research. For more details, see the copy of the consent form at the end of this article. This trial does not involve collecting biological specimens.

## Interventions

### Explanation for the choice of comparators {6b}

A combined cognitive training programme (CCTP) was chosen as a comparator to study the most significant effect potential concerning a traditional cognitive training programme (TCTP) in treating patients with MCI.

### Intervention description {11a}

In the CCTP, the patients will be organised into groups composed of three participants. The patients will realise a combined electrical stimulation and cognitive training programme. That contemplates 12 WM training sessions based on tasks of storage and manipulation of verbal and visuospatial information, with a frequency of twice a week for 6 weeks, completing a total of 12 sessions in presential format. Concomitantly, the participants will receive real electrical stimulation for 10 min during a verbal working memory task. The intervention will be delivered by trained personnel in this technique and an assistant to groups. This electrical stimulation protocol includes 10-s periods of phase-in and 10 s of phase-out and will be done in parallel with the cognitive intervention programme in sessions 3, 4, 5, 6, 7, 8, 9, and 11. The electrical stimulation will be applied with two 3 × 1 arrays of electrodes. The central stimulation electrodes will be positioned in F3 and CP3 (according to the international 10–20 system). The AC stimulation will be one mA from the baseline to the stimulation peak. The stimulation will have a gamma sine waveform (80 Hz) over the positive phase of the theta oscillation[33] individualised for each patient ranging from 4 to 8 Hz and the phase-locked between arrays [36, 37]. The electrode impedances will always be maintained under 10 kOhms.

Participants in the control group (TCTP) will start a cognitive intervention programme identical to the treatment group and will be organised into groups of three participants. Still, unlike the treatment group, they will receive placebo electrical stimulation, i.e. an electrical current will be applied for a short period at the beginning of the session (30 s). Then, the electric stimulation will be stopped. This procedure induces a sensation in the skin similar to real electric stimulation without generating a detectable impact on the brain state. They will be performed parallel to the cognitive intervention programme in sessions 3, 4, 5, 6, 7, 8, 9, and 11.

### Criteria for discontinuing or modifying allocated interventions {11b}

Participants can withdraw at any point if a participant in either arm indicates that they wish to discontinue the trial. In this case, they will only be contacted further by the research team to invite them to take part in a brief written survey to ascertain their reasons for not taking part. However, the participants will have no intermediate levels of discontinuation of the trial.

We will assess whether it is in the participant’s best interest to continue or discontinue trial treatment if a serious adverse reaction occurs. In such cases, discontinuation of the intervention will be recommended. If an unexpected serious adverse reaction is linked directly to trial participation or treatment, the trial will be temporarily halted for investigation. If future risk cannot be mitigated, the trial will be discontinued. The sponsor, ethics committee chair, and principal investigator will lead this process. The same protocol will be followed if information surfaces indicating that the therapy intervention or trial procedures are unsafe.

### Strategies to improve adherence to interventions {11c}

Individual interviews at the beginning of both programmes (CCTP and TCTP) will reinforce the research rationale, highlight the importance of assistance to all treatment sessions and following, and identify potential barriers to participation. We will offer training to help patients familiarise themselves with the technical aspects of computers utilised in the cognitive programmes. We will call to remind each patient before the appointment and will provide transportation to assist on site.

### Relevant concomitant care permitted or prohibited during the trial {11d}

All patients will be encouraged to continue medical controls and treatments as usual.

### Provisions for post-trial care {30}

The patients’ clinical teams will be informed in writing of their participation at the end of the trial. There is no anticipated harm or compensation for trial participation.

### Outcomes {12}

#### Primary outcome

The primary result will be prefrontal theta oscillatory activity. This continuous variable is sensitive to electrophysiological changes underlying cognitive processes as WM. It has been described in several works of our group and others [28, 38–43]. The primary timepoint for outcome measures will be three times: pre-intervention, 1 and 12 weeks post-intervention. This outcome corresponds to the ratio of the normalised power (mean/standard error, mV^2^) of the prefrontal theta oscillation (5–10 Hz, F3 electrode) related to successful memory performance (SMP). This prefrontal theta oscillation is calculated based on a single-trial general linear model that includes SMP as the regressor of interest and memory load as a secondary regressor.

#### Secondary outcomes

Secondary results will correspond to performance measures in validated test work memory tasks:

The secondary timepoint for outcome measures will be three times: pre-intervention, 1 and 12 weeks post-intervention.

(1) WAIS-IV Digit Retention subtest. Chilean standard [44]

It is part of the WM Index and consists of three tasks: (a) Direct Digits: repeat a series of digits, presented orally, in the same order as they are presented; (b) Reverse Order Digits: repeat a series of digits in reverse order as they are presented; and (c) Sequence Digits: order and repeat from lowest to highest a series of numbers read by the examiner. These tasks assess attention and resistance to distraction, immediate auditory memory, and WM.

(2) Trail Making Test B (TMT-B). Chilean standard [45]

This part of TMT reflects mainly WM and, secondarily, the capacity for cognitive flexibility [46]. It connects 25 numbers and letters in ascending order, alternating between them (e.g. 1-A, 2-B, 3-C, etc.). The score is obtained when it takes an individual to complete the task.

(3) Parietal theta oscillatory activity. The ratio of the normalised power (mean/standard error, mV^2^) of parietal theta oscillation (5–10 Hz, CP3 electrode) related to memory load. This parietal theta oscillation is calculated based on a single-trial general linear model that includes memory load as the regressor of interest and successful memory performance (SMP) as a secondary regressor.

#### Baseline survey

Participant characteristics will be assessed as part of the initial evaluation and clinical interview to identify eligible patients who will be invited to participate in the trial. Once selected, participants who have agreed to participate in the trial will be evaluated 1 week before the randomisation with electrophysiological measures and performance in validated test work memory tasks:

### Participant timeline {13}

**Fig. 1:**
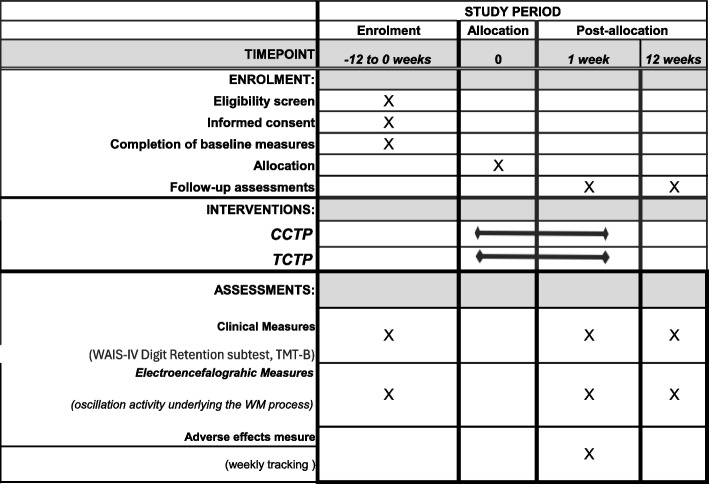
Schedule of enrollment, interventions, and assessments (displayed according to Standard Protocol Ítems: Recommendations for Interventional Trials [SPIRIT])

Potential participants who express interest in the study will undergo a structured on-site interview with the researcher to determine their eligibility for participation. Those who meet the initial screening criteria will be invited for an on-site assessment session, during which they will undergo structured clinical interviews to confirm their eligibility for the study. Eligibility interviews will be conducted immediately after the initial screening. Patients who agree to participate in the trial will then undergo baseline assessments of their electrophysiological measures underlying WM processes, as well as performance measures in validated WM tasks, including the WAIS-IV Digit Retention subtest [44] and the Trail Making Test B (TMT-B) [45]. Baseline assessments will be conducted within 1 week before randomisation. Participants will be randomly allocated and given at least 1 week to become familiarised with the computational devices before the intervention period, which will last 6 weeks. After the intervention period, participants will be assessed again at 1 week and 12 weeks post-intervention. Please refer to Fig. 1 for a schematic diagram of the enrollment, intervention, and assessment schedule.

### Sample size {14}

For the estimation of the minimum required sample size, we will consider the following parameters: (a) Effect size for the mixed ANOVA statistical test (2 × 2, with interaction effects), (b) Statistical power (1 − *β*) = 0.95 and (c) Significance level α = 0.05. We will use the G*Power 3 software to calculate [47]. Considering an effect size *η*^2^ = 0.06, the sample size amounts to 54 participants (n1 = 27; n2 = 27). As our study will be a two-intervention effect, we will consider an effect size somewhat smaller than those observed for comparisons between clinical populations of prefrontal theta oscillations during working memory: for example, Lenartowicz: *η*^2^ = 0.09 [38], and which coincides with the work of our team *η*^2^ ~ 0.1 [28]. The effect size we will use to calculate the sample is considered a moderate to significant effect according to Cohen’s criteria [48]. However, the initial sample size we propose in this project finds the proportion of participants who may present low adherence to the study. In this regard, we can point out that in a preliminary study with similar characteristics, a drop-out rate of about 15% was observed. Therefore, considering the drop-out percentage and the minimum sample size required to investigate an effect, the initial sample will be 62 participants (n1 = 31; n2 = 31).

### Recruitment {15}

**Fig. 2:**
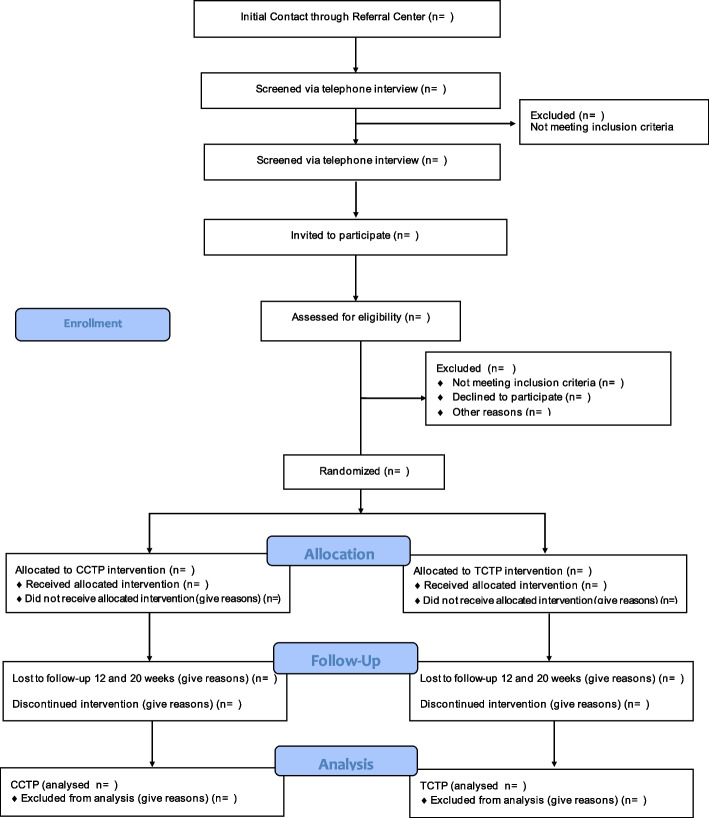
CONSORT diagram describing the flow of participants through the study

Participants, non-institutionalized users with mild cognitive impairment, will be recruited from the Center of Phonoaudiological Attention of the University of Valparaíso (CAFUV) from Valparaiso (Chile). The recruitment of participants will be done by a clinical team consisting of a neuropsychologist, a clinical psychologist, and speech therapists, who will apply the instruments to corroborate the fulfilment of the inclusion–exclusion criteria. In addition, they will carry out the evaluations and interventions that the participants will receive once these users have expressed their willingness to participate in the study by signing informed consent (see Fig. 2).

## Assignment of interventions: allocation

### Sequence generation {16a}

Individual participants will be allocated to either CCTP or TCTP at a 1:1 ratio through remote randomisation by the staff responsible for randomisation at the Biomedical Informatics Center Clinica Alemana—Universidad del Desarrollo, Chile (CIMB) (https://redcap.cibm.cl). The clinical trial will use the stratified randomisation method, with age (< 75 years and ≥ 75 years), sex (male and female), and years of schooling (≤ 12 years of education and > 12 years of education) as the stratification variables. Randomisation will be performed under the control of a blinded worker from CIMB, who is the only person allowed to manage the electronic coding of the randomisation to assign the individuals. The researchers will be blind to the group to which the participant is allocated.

### Concealment mechanism {16b}

In order to perform the allocation concealment process, the coded groups will be placed in sealed opaque envelopes, each marked with a participant’s code, and held by the staff responsible for randomisation. The envelopes will only be opened before the cognitive training session. To ensure proper blinding, participants will receive a code and will be shielded from the allocation process by independent CIMB staff responsible for randomisation. While the experimenter administering the intervention will be aware of the group allocation, those conducting cognitive and electrophysiological pre- and post-evaluations will remain blinded to participants’ group allocations.

### Implementation {16c}

The sealed envelopes will be opened and concealed again in the first cognitive training stage prior to the intervention by the staff responsible for randomisation. Then, the code will inform the cognitive training experimenter.

## Assignment of interventions: blinding

### Who will be blinded {17a}

The person responsible for generating the random assignment sequence list must be made aware of the people who recruit and evaluate the participants. This generated sequence will be blind until the interventions are assigned. The type of masking will be double-blind since both the participants and the professionals who perform the pre-post evaluation will. For technical reasons, professionals administering electric interventions are not blinded to the type of electrical stimulation (i.e. real or placebo) being applied. The statistician analysing outcome data will remain blind to treatment allocation throughout the analysis, which will be conducted with groups indicated by an anonymised code.

### Procedure for unblinding if needed {17b}

In the event of an adverse reaction by a participant in either treatment arm, unblinding may become necessary. Researchers will only be unblinded if knowledge of the treatment arm is deemed essential to the patient’s management by their clinical team. Any instances of unblinding will be documented, but we anticipate that they will not have a biasing effect on follow-up assessments.

## Data collection and management

### Plans for assessment and collection of outcomes {18a}

At baseline, trained researchers will evaluate the eligibility throughout a clinical semi-structured interview and application of the following scales:

1. Montreal Cognitive Assessment (MOCA) Chilean Norm: Screening test to evaluate the general cognitive state and detection of mild cognitive impairment (MCI) elaborated by [49]. Assessment of cognitive functions, attention, abstraction, memory, language, visuospatial and visuoconstructive capabilities, calculation, and orientation.
2. Technology-Activities of Daily Living Questionnaire (T-ADLQ) Chilean Norm [50]. The T-ADLQ assesses functional abilities of daily living in persons with MCI and the probable evolution to dementia. It is answered by the informant who accompanies the person with MCI/Dementia. The T-ADLQ measures self-care and personal care in the following areas: personal care, home care, employment and recreation, shopping and money management, travel and communication, and the use of technology.
3. Geriatric Depression Scale of Yesavage (5-GDS). Chilean Norm [51]: An instrument widely used for depression screening. There is an abbreviated 5-item version, validated in 2000 for the Chilean population.
4. Hopkins Verbal Learning Test-Revised. (HVLT-R). Chilean Norm [45]: Verbal learning test, which assesses anterograde memory using the immediate and delayed word repetition paradigm. In addition, it includes a recognition phase.
5. INECO Frontal Screening (IFS-Ch) Chilean Norm [52]: The screening test that evaluates several executive processes. In particular, it evaluates motor programming, resistance to interference, inhibitory control, verbal working memory, spatial working memory, and abstraction/conceptualisation.

Later, trained researchers will realise pre-treatment and follow-up assessments at 8 and 20 weeks. They will assess electrophysiological measures underlying working memory (WM) processes, as well as performance measures in validated WM tasks, including the WAIS-IV Digit Retention subtest [44] and the Trail Making Test B (TMT-B) [45].

### Plans to promote participant retention and complete follow‐up {18b}

A researcher assistant will call participants to remember the on-site sessions (evaluations, intervention, and follow-up) and provide them with transportation for each session.

### Data management {19}

Data management will be provided by the Clinical Studies Center of Medicine Faculty of the Clínica Alemana-Universidad del Desarrollo. Interventions will be delivered on site by researchers and therapists at the Center of Phonoaudiological Attention of the University of Valparaiso (CAFUV) in collaboration with researchers of Social Neuroscience and Neuromodulation Laboratory (neuroCICS), Universidad del Desarrollo, Santiago, Chile. The CAFUV will include further patient identification centres (PICs) where needed and helpful. Potential PICs will need to be able to recruit a considerable number of patients (for more detail, see Sect. 15. Recruitment).

The Biomedical Informatics Center at Clinica Alemana-Universidad del Desarrollo in Chile (https://redcap.cibm.cl) will oversee randomisation, data management, and quality assurance under the supervision of the CI and quality assurance manager. The local ethical committee hosting the research will store routine clinical notes according to standard practice. The research team will enter assessment data on a secure, web-based system maintained by the Biomedical Informatics Center. Consent forms will be stored separately from data, and data will be anonymised wherever possible. Datasets generated and analysed during the trial will be stored in a non-publicly available repository at the Biomedical Informatics Center upon publication of the main study results. Anonymised data may be accessed and analysed by members of the project team and collaborating researchers. All personally identifiable data, except for consent forms, will be destroyed as soon as the study closes unless participants have consented to be contacted for future research, in which case their contact details will be kept for 5 years. Research data with personal information removed and replaced through a code will be retained for 5 years before destruction. Electronic records will be held for 5 years after the end of the study. Publications will not contain any patient-identifiable information.

### Confidentiality {27}

The trial data collected will be strictly confidential within the research team and services involved. Confidentiality will only be broken in exceptional circumstances when the researcher or therapist deems immediate risk is present to a patient or someone else, and the clinical team or other relevant professionals must be contacted. All data will be stored and processed under the local Ethical Committee guidelines. Personal data will be link-anonymised and identified by a code known only to the research team. Names and contact details will be stored in password-protected files on secure servers and kept separate from link-anonymised data.

### Plans for collection, laboratory evaluation and storage of biological specimens for genetic or molecular analysis in this trial/future use {33}

As described under 26b, there will be no biological specimens collected.

## Statistical methods

### Statistical methods for primary and secondary outcomes {20a}

Both the intention-to-treat (ITT) analysis set and the per-protocol (PP) analysis set will be utilised. The ITT set comprises all participants who have been randomised. When a participant is randomly assigned to a combining cognitive training group, they should be included in the combining cognitive training analysis. For the PP set, only participants who complied with combining cognitive training will be analyzed. The ITT analysis will be compared with the PP analysis to ascertain whether the two results are consistent.

#### Inferential analysis

Compliance with assumptions for parametric testing will be contrasted. In particular, univariate normality (i.e. Kolmogorov-Smirnoff, Shapiro–Wilk), sphericity (i.e. Mauchly’s W), and homoscedasticity of error variances (i.e. Levene test). If any of the above assumptions are violated, the corresponding non-parametric estimates will be provided. Mixed-model analyses without any ad hoc imputations will be used to handle missing data in case of participant drop-out and to conduct the efficacy analysis [53]. To assess distributional assumptions and the impact of missing random effect components on the model, several tests will be conducted. Normality assumptions will be examined using the Shapiro–Wilk test, skewness and kurtosis indices, and normality plots (Q-Q plots). Homoscedasticity will be investigated using Levene’s test and Box’s M test for equivalence of covariance matrices. The Wald statistic (or *Z*-test) will be utilised to test residual error variance estimation and the null hypothesis of homogeneity of residuals. The assumption that data is missing completely at random (MCAR) will be assessed using Little’s MCAR test, which remains robust to violations of distributional assumptions [54]. Additionally, the primary outcome will be evaluated using Bayesian hierarchical approaches employing JAGS and R software. In this approach, each individual beta per regressor will be subjected to a population distribution of beta values that follow a normal distribution [55]. The prior assumption for each population distribution of beta values will be flat (non-informative). The correct convergence of the Markov Chain Monte Carlo (MCMC) will be evaluated using the Gelman-Rubin statistic. The model fit will also be assessed using DIC (Deviance Information Criterion) and LOOIC (Leave-One-Out Information Criterion).

The effect sizes will be reported for each electrophysiological and cognitive performance measure analysis through the index η^2^p (partial eta square). The respective post hoc comparisons will use Bonferroni’s correction, and the effect size will be reported through Cohen’s index *d*. Graphs of the interaction effects will be provided if observed. In case of transgression of the sphericity assumption, the Greenhouse–Geisser correction will be made. Consequently, the respective parametric statistics will be provided (i.e. Friedman test, Connover’s post hoc tests). For all hypothesis tests, a significance level will be considered *⍺* = 0.05.

### Interim analyses {21b}

A committee focused on data monitoring and ethics will regularly review outcome data throughout the data collection process.

### Methods for additional analyses {20b}

We do not intend to conduct subgroup analyses.

### Methods in analysis to handle protocol non‐adherence and any statistical methods to handle missing data {20c}

The statistical analyses, including mixed models and Bayesian estimation, will be conducted without resorting to ad hoc imputations to manage missing data resulting from participant drop-out and to perform efficacy analysis. Little’s MCAR test will determine whether the missing data are completely at random, ensuring the robustness of the analyses even without data imputation.

### Plans to give access to the full protocol, participant‐level data and statistical code {31c}

The sharing of data will be facilitated through a controlled access model, which adheres to the guidelines set forth by the Ethical Committee and recommendations provided by the Biomedical Informatics Center Clinica Alemana—Universidad del Desarrollo in Chile (https://redcap.cibm.cl). To gain access to the data for future projects, scientists must submit a formal request to the CI, and any project utilising the data must have received approval from the Ethical Committee and regulatory bodies overseeing the release of anonymised data. We will strictly adhere to current recommendations regarding anonymisation and curation of trial data for sharing purposes [56].

## Oversight and monitoring

### Composition of the coordinating centre and trial steering committee {5d}

The CI will assume responsibility for the overall management of the trial and delivery of the work. The CI will lead the core research team (including contact with the ethical committee, the trial manager, and research assistants), which will meet monthly via videoconference and presential meetings and receive input from the broader research group. The Clinical Studies Center of Medicine Faculty at Universidad del Desarrollo (Chile) will monitor all aspects of the conduct and progress of the trial, ensure that the protocol is adhered to, and take appropriate action to safeguard participants and the quality of the trial.

### Composition of the data monitoring committee, its role, and reporting structure {21a}

A Data Monitoring Committee (DMC) was deemed unnecessary due to the minimal risk posed to participants and the trial’s small scale.

### Adverse event reporting and harms {22}

#### Risk monitoring

To identify potential risk issues, our research assistants will conduct a screening every 48 h after the completion of the intervention sessions. This screening will involve checking the skin condition on the skull where the electrodes were placed and mild headache. These cautions are important to ensure no adverse effects or complications from the intervention and mitigate potential risks to the participants.

#### Adverse event and serious adverse event recording and reporting

Possible adverse events and other unintended effects of the trial will be documented in the medical records and periodically reported to the Scientific Ethics Committee. The expected hazards in this trial, according to safety guidelines, were skin damage on the skull where the electrodes were placed and mild headache. If unexpected events occur, we will use MedDRA in the trial publication to report all adverse events. Lethal or severe adverse events will be reported to the Health Services of the Region of Valparaíso (Chile) and the Scientific Ethics Committee as soon as possible or within 7 days of getting informed of the adverse event.

### Frequency and plans for auditing trial conduct {23}

The research will be audited biannually through the procedures established at the Clinical Studies Centre of the Faculty of Medicine of the Clínica Alemana—Universidad del Desarrollo (Chile).

### Plans for communicating important protocol amendments to relevant parties (e.g. trial participants, ethical committees) {25}

If any changes to the protocol become necessary, we will seek sponsor approval for the proposed amendments before submission. We will then prepare a request to the Research Ethics Committee through our local system (https://medicina.udd.cl/comite-etico-cientifico/), authorised by both the CI and the sponsor. The CI will promptly communicate the outcomes of the review process and any resulting protocol modifications to the participating sites and relevant organisations. Furthermore, we will update the trial registrations and the published protocol to reflect these amendments accordingly.

### Dissemination plans {31a}

The trial data has the potential to shape changes in the current approach to addressing MCI and to slow down the progression of Alzheimer’s dementia. The assessment of treatments for patients will further facilitate the training and dissemination of this approach, not only within MCI but also in other relevant contexts. Insights gained from qualitative analyses will provide valuable information regarding its feasibility. The research findings will be disseminated widely and published in peer-reviewed scientific journals and professional publications. We will also present our results at conferences and workshops and, when feasible, share our findings through various media and social media platforms. Additionally, we are committed to sharing these findings at the local level with participants, healthcare services and other stakeholders.

## Discussion

Globally, a notable sociodemographic transformation is in progress, primarily defined by an ageing population and changes in the epidemiological profile, leading to a heightened prevalence of chronic diseases and neurological conditions, including dementia and mild cognitive impairment (MCI). These conditions carry substantial socioeconomic implications for patients and indirectly impact their families and society.

MCI can be viewed as an early stage of certain dementias, making it a crucial point for implementing interventions aimed at delaying cognitive decline, reducing dependence, and preserving the quality of life. A critical cognitive facet open to intervention is working memory, a fundamental element of daily executive functioning and a gateway to long-term memory preservation. Consequently, there is growing interest in assessing the impact of specific interventions in this cognitive domain.

One promising approach to address this challenge involves combining techniques that have individually demonstrated efficacy in enhancing memory performance [57, 58]. As such, this project proposes a range of cognitive training methods and NIBS techniques. Both strategies have shown positive effects in older adults, supporting that the ageing brain can be selectively and enduringly trained [59–62]. Specifically, we will investigate the effects tACS in conjunction with personalised and progressive cognitive training [33, 36, 63–65].

For instance, Grover et al. [36] illustrated the repetitive use of transcranial alternating current stimulation (tACS) over 4 days, leading to a sustainable improvement in auditory-verbal working memory and long-term memory in individuals aged 65 to 88. Notably, the modulation of synchronous low-frequency activity in the parietal cortex resulted in a preferential enhancement of working memory on the third and fourth days, as well as 1 month after the intervention.

In summary, this project aims to provide insights into the combined impact of cognitive training and transcranial alternating current stimulation on MCI outpatients. Regarding this neuromodulation technique, the study seeks to delineate highly localised spatial-spectral parameters of memory-specific cortical circuitry, equipping clinicians with practical tools to intervene in MCI patients.

## Trial status

The project officially started on January 5, 2019, with Consent Version No. 3 being signed on October 30, 2019. However, recruitment was delayed until January 26, 2022, owing to the impact of the COVID-19 pandemic. We project the recruitment phase to conclude by January 26, 2024, with the overall study ending on December 30, 2024.

## Funding {4}

This trial is funded through the National Fund for Health Research and Development in Health, Chile (FONIS for its acronym in Spanish) of the National Research and Development Agency (ANID), Moneda #1375 Santiago of Chile. The funder’s number for this trial is SA19I0118.

## Availability of data and materials {29}

The datasets produced and analyzed during the present study are not publicly accessible due to the ongoing status of the protocol at the time of submission. The Clinical Investigator will serve as the custodian of the trial data, and any data necessary to support the protocol can be provided upon request.

### Supplementary Information

**Additional file 1.** Consent form (original)

## Abbreviations

CAFUV: Center of Phonoaudiological Attention of the University of Valparaiso
CCTP: Combined cognitive training programme
MCI: Mild cognitive impairment
NIBS: Non-invasive brain stimulation
PI: Principal investigator
SMP: Successful memory performance
tACS: Transcranial electrical stimulation of alternating current
TCTP: Traditional cognitive training programme
tDCS: Transcranial direct current stimulation
TMT-B: Trail Making Test B
WM: Working memory
