# Supplementary material for: The effect of a cognitive training therapy based on stimulation of brain oscillations in patients with mild cognitive impairment in a Chilean sample: study protocol for a phase IIb, 2 × 3 mixed factorial, double-blind randomised controlled trial
Source: Trials. 2024 Feb 23;25:144. doi: 10.1186/s13063-024-07972-7 (PMC10885461; doi:10.1186/s13063-024-07972-7)
Supplement: Supplementary file 1 — Additional file 1. Consent form (original) [file 13063_2024_7972_MOESM1_ESM.pdf]

**Informed Consent**

The purpose of providing the following information is to assist you in making the decision to participate or not in this project. This "Informed Consent" will be read together with the participant in an interview conducted by at least one of the project's responsible researchers to clarify any doubts and enable an informed decision regarding the implications of participating or not in the proposed research. Participants will be given the option to take the "Informed Consent" with them to consult with their family or other individuals they consider appropriate before making a decision regarding their participation in the project.

Title of the Research Project:

**"Evaluation of a Cognitive Training Therapy Based on Brain Oscillation Stimulation in Patients with Mild Cognitive Impairment through a Randomized Clinical Trial"**

FONIS Project Folio SA19I0118


Principal Investigator of the Study:

Pablo Billeke Bobadilla M.D. Ph.D.

Social Neuroscience and Neuromodulation Laboratory (neuroCICS)

Center for Research in Social Complexity (CICS)

School of Government, Universidad del Desarrollo, Chile

Av. Las Condes #12461, Tower 3, Office #307, Las Condes, Santiago

Tel. 02-232 79 636

pbilleke@udd.cl

http://neuroCICS.udd.cl

**Research Objectives:**

The aim of this research is to evaluate the effect of a combined intervention involving cognitive training and brain electrical stimulation in a group of individuals with Mild Cognitive Impairment compared to an intervention involving only cognitive training.

Brain electrical stimulation is a widely used clinical technique that safely and non-invasively allows the stimulation of certain regions of the brain by applying low-intensity electric current to the patients' scalp. The most commonly reported adverse effect is a mild discomfort (irritation or itching) in the application area of the current, and if the discomfort persists, the session will be immediately suspended. The type of current you may receive can be real (providing actual electric current) or placebo (simulating the perceived sensation but not actually applying electric current).

On the other hand, cognitive training will involve the completion of tasks in front of a computer in a room equipped for three people, accompanied by a monitor who will guide and resolve any queries. These tasks have been specially designed to train the cognitive ability of working memory (the capacity that allows us to maintain and temporally manipulate information to guide our behavior towards a specific goal).

All procedures will take place at the Center for Speech and Hearing Care of the University of Valparaíso (CAFUV).

**PROCEDURE DETAILS:**

Participation in this project involves four stages, which are detailed in the following outline:

Study Outline

| | **Study Groups** | |
|---|---|---|
| **Project Stages** | Group 1 | Group 2 |
| Pre-treatment Assessment<br><br>(1 hour) | a. Electroencephalographic assessment with working memory tasks<br><br>b. Cognitive assessment of working memory | a. Electroencephalographic assessment with working memory tasks<br><br>b. Cognitive assessment of working memory |
| Cognitive Intervention<br><br>(45 minutes) | Cognitive Training<br>+<br>Brain Stimulation<br>real | Cognitive Training<br>+<br>Brain Stimulation<br>placebo |

| | | |
|---|---|---|
| Post-treatment Assessment<br><br>(1 hour) | a. Electroencephalographic assessment with working memory tasks<br><br>b. Cognitive assessment of working memory | a. Electroencephalographic assessment with working memory tasks<br><br>b. Cognitive assessment of working memory |
| 3 Months Post-Treatment Assessment<br><br>(1 hour) | a. Electroencephalographic assessment with working memory tasks<br><br>b. Cognitive assessment of working memory | a. Electroencephalographic assessment with working memory tasks<br><br>b. Cognitive assessment of working memory |

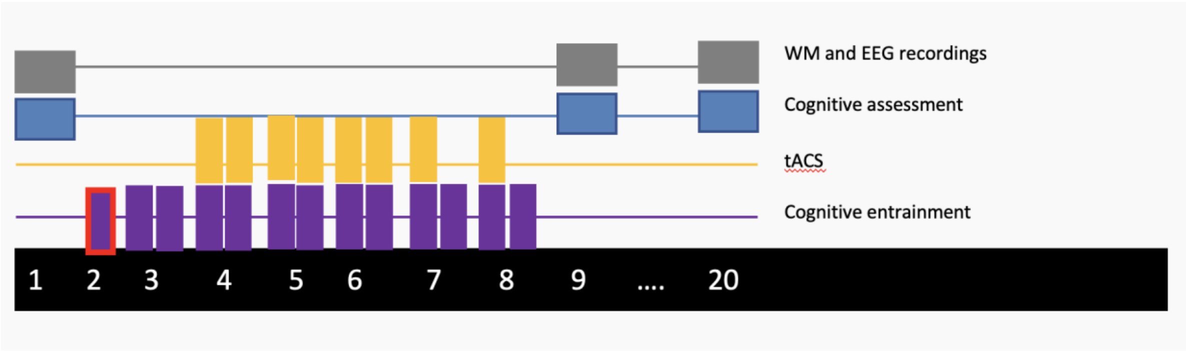


**BENEFITS OF THE STUDY:** As a participant in this research, you will not receive a direct benefit. Instead, you will contribute to a better long-term understanding of this condition and new treatment alternatives.

**RISKS:** All procedures in this research involve no risks for the participants. The most common adverse reaction is a mild discomfort (irritation or itching) in the area of application of the electrical brain stimulation. If the discomfort persists, the session will be immediately suspended.

**COSTS AND COMPENSATION:** All evaluations and procedures included in the study will be free of charge. Participants will be compensated for transportation expenses at the end of each session. Although no risks associated with the applied procedures have been described, in the event of any adverse event directly related to the application of this protocol, the participant will be referred to a consultation with the neurologist associated with the project. This appointment will not incur any costs for the participant, and any doubts will be resolved, and guidance will be provided for the continuity of treatment at the appropriate health facility, if necessary. These costs will not be covered by the funds of this project.

**DATA RETURN:** At the end of the study, a descriptive and non-diagnostic report will be delivered to the participant in person.

**CONFIDENTIALITY:** Although the results obtained are intended to be published in scientific journals, the identity of each participant will be kept confidential and coded. Any person not involved in this research lacks access to information that would identify the volunteers participating in this study. The data obtained will be stored digitally, in duplicate, on different media (hard disk and DVD as a backup) for the duration of the study at the Center for Social Complexity Research of the Universidad del Desarrollo. Once the study is completed, the data will be stored only on solid media (DVD) on the university premises, to have a backup for the eventual verification of results and analysis procedures.

It is possible that the data collected in the context of this research may be used in subsequent studies that benefit from the type of records obtained. If this is the case, only coded data will be available, maintaining the identity of each participant strictly confidential.

**PARTICIPATION IN THE STUDY:** The decision to participate in this study is entirely voluntary and personal. Granting consent as a participant will have no impact on the regular treatment you receive. Similarly, if you were to feel uncomfortable and wish to withdraw from the study, you are free to do so at any time and without any consequences.

**SAMPLE SELECTION:** The selection of participants will be conducted by the clinical team at the Center for Speech Therapy Care at the University of Valparaíso (CAFUV), based on users with Mild Cognitive Impairment referred to the center.

**COMMUNICATION WITH THE PRINCIPAL INVESTIGATOR:**

If you have any doubts or questions about this research, you can contact the responsible investigator:


Pablo Billeke Bobadilla

Laboratory of Social Neuroscience and Neuromodulation

Center for Research in Social Complexity

Faculty of Government, Universidad del Desarrollo, Chile

Av. Las Condes #12461, Tower 3, Office 307, Las Condes, Santiago

Tel. 02-232 79 636. pbilleke@udd.cl


Approval: This study has been approved by the Scientific Ethics Committee of the Universidad del Desarrollo. In case of any inquiries about your rights as a participant in the study or in the event of any potential conflicts, you can contact Dr. Marcial Osorio, President of the Scientific Ethics Committee of the Universidad del Desarrollo (Faculty of Medicine UDD / Clínica Alemana de Santiago, Avenida Las Condes #12461, Tower 3, 2nd floor, Las Condes, Santiago de Chile. Phone (562) 3279157, e-mail: ceccasudd@udd.cl).

If you agree to participate in this study, you must sign two copies of this Informed Consent. One copy will be for the investigator who explained each of the points of the research, and the other copy is for you to keep once signed.

## DECLARATION OF THE PARTICIPANT

In the present study, _______________________________________________ (name of the informing researcher) has clearly explained to me, both verbally and in writing, that I will be participating in the research project titled "Evaluation of a cognitive training therapy based on brain oscillation stimulation in patients with Mild Cognitive Impairment through a randomized clinical trial" with the identification code FONIS Folio SA19I0118. I understand that my participation will include a cognitive assessment of performance in working memory tasks, an electroencephalographic recording, and a cognitive intervention program along with electrical brain stimulation, either real or placebo. Furthermore, I am aware that each phase of the study will have a maximum duration of 60 minutes.

Considering all of the above, I _______________________________________________ voluntarily confirm my participation in this research. Likewise, I affirm that I have had the opportunity to clarify all my doubts, and that Dr. Pablo Billeke Bobadilla has expressed his availability to assist me should any additional questions arise. Also, I acknowledge that if I feel uncomfortable and wish to withdraw from the study, I am free to do so at any time and without any consequences.

**Participant's name:** _________________________________________________________

**Participant's signature:** ___________________________________________________________

Participant's ID number: ____________________________________________________________

Signature of the Informing Researcher:

______________________________________________________

**Signature of the Responsible Researcher:** Dr. Pablo Billeke Bobadilla

Date and time: ___________________________________________________________________

**Signature of the Institution's Representative:**

_______________________________________________

Name: Carlos Rodríguez S.

Date and time: ___________________________________________________________________
